# Process inhomogeneity leads to rapid side product turnover in cultivation of *Corynebacterium glutamicum*

**DOI:** 10.1186/1475-2859-13-6

**Published:** 2014-01-10

**Authors:** Friedrich Käß, Stefan Junne, Peter Neubauer, Wolfgang Wiechert, Marco Oldiges

**Affiliations:** 1 Institute of Bio- and Geosciences, IBG-1: Biotechnology, Systems Biotechnology, Forschungszentrum Jülich GmbH, Jülich D-52425, Germany; 2 Chair of Bioprocess Engineering, Department of Biotechnology, Technische Universität Berlin, Ackerstrasse 71-76, Berlin D-13355, Germany

**Keywords:** Scale-down, Oxygen supply limitation, Two-compartment reactor, STR-PFR, Oxygen uptake redistribution, Metabolic robustness

## Abstract

**Background:**

*Corynebacterium glutamicum* has large scale industrial applications in the production of amino acids and the potential to serve as a platform organism for new products. This means the demand for industrial process development is likely to increase. However, large scale cultivation conditions differ from laboratory bioreactors, mostly due to the formation of concentration gradients at the industrial scale. This leads to an oscillating supply of oxygen and nutrients for microorganisms with uncertain impact on metabolism. Scale-down bioreactors can be applied to study robustness and physiological reactions to oscillating conditions at a laboratory scale.

**Results:**

In this study, *C. glutamicum* ATCC13032 was cultivated by glucose limited fed-batch cultivation in a two-compartment bioreactor consisting of an aerobic stirred tank and a connected non-aerated plug flow reactor with optional feeding. Continuous flow through both compartments generated oscillating profiles with estimated residence times of 45 and 87 seconds in the non-aerated plug flow compartment. Oscillation of oxygen supply conditions at substrate excess and oscillation of both substrate and dissolved oxygen concentration were compared to homogeneous reference cultivations. The dynamic metabolic response of cells within the anaerobic plug flow compartment was monitored throughout the processes, detecting high turnover of substrate into metabolic side products and acidification within oxygen depleted zones. It was shown that anaerobic secretion of lactate into the extracellular culture broth, with subsequent reabsorption in the aerobic glucose-limited environment, leads to mixed-substrate growth in fed-batch processes. Apart from this, the oscillations had only a minor impact on growth and intracellular metabolite characteristics.

**Conclusions:**

Carbon metabolism of *C. glutamicum* changes at oscillating oxygen supply conditions, leading to a futile cycle over extracellular side products and back into oxidative pathways. This phenomenon facilitates a dynamic and flexible shift of oxygen uptake at inhomogeneous process conditions. There is no loss of process characteristics at oscillation times in the minute range, which emphasizes the robustness of *C. glutamicum* in comparison to other industrial microorganisms. Therefore, the metabolic phenotype of *C. glutamicum* seems to be particularly well-suited for cultivation at inhomogeneous process conditions for large-scale fed-batch application, which is in good accordance with the respective industrial experiences.

## Background

*Corynebacterium glutamicum* is an important organism for industrial biotechnology. Application in large scale amino acid production is state of the art in the food and feed industries, with several established amino acid products [1]. Currently, millions of tons of amino acids for food and feed application (*e.g.* continuously improving L-lysine strains [2], *etc.*) are produced using *C. glutamicum* every year. Also, the increasing demand for bio-based fine-chemicals is fuelling the search for new and efficient platform organisms, with *C. glutamicum* as one promising candidate (*e.g.* succinate production [3], L-valine [4-6], 1,2-propanediol [7], L-alanine [8], and other organic acids [9,10]). Typically, industrial products of *C. glutamicum* are bulk chemicals produced in mostly aerobic processes using reactors of up to 500 m^3^. Due to the high metabolic activity of microorganisms, cultivation is performed in fed-batch mode in stirred tank reactors. The limited carbon source feeding reduces oxygen demand and heat generation. This is beneficial in large scale reactors, which are often restricted in these aspects due to technical and commercial limitations.

A direct consequence of large scale cultivation is increased gradient formation of nutrients and environmental process parameters (*e.g.* pO_2_, pH, pCO_2_, substrate concentration), which represents a major challenge in industrial biotechnology [11]. Gradients are caused by insufficient mixing, combined with the high metabolic activity of microbial cells. One example is oxygen supply distribution: the solubility of oxygen is very low at typical bioprocess conditions, and therefore oxygen is rapidly depleted in zones of low aeration. An individual microorganism within a large-scale culture is exposed to gradients and oscillating changes of its environment in terms of substrate availability and dissolved oxygen. Depending on parameters such as mixing or homogeneity time, *i.e.* time required for a defined depletion of gradients [12], oscillations can vary in their duration and statistical distribution for individual cells in a microbial culture.

In contrast to large scale bioreactors, gradients and inhomogeneities are rarely observed under lab-scale conditions. Most procedures for strain selection during screening and early process development are carried out with shaken or stirred tank bioreactors in a volume range of milliliters to liters, which is typically associated with mixing times in the range of seconds [13,14]. Depending on the final production scale, industrial homogeneity times of up to several minutes can be expected for typical bioprocesses [12], which results in substantial gradients and an oscillating exposure of the microbial cells to these gradients. This constitutes a pitfall for scale-up of microbial processes, since strain choice and process engineering are derived from well-mixed laboratory experiments. As a result, selected strains can show decreasing performance during sequential scale-up, *i.e.* increasing homogeneity time and oscillation, resulting in time- and cost-intensive additional strain or process iteration. Procedures for evaluating metabolic robustness against oscillations can therefore facilitate the selection of robust strains during laboratory development, and can improve transferability of processes from laboratory to production scale [15,16].

Scale-down simulators are a promising tool for investigating the metabolic impact of industrial production conditions. They are gaining increasing attention, as has been shown in recent perspective publications [17,18]. They are mostly used to generate oscillating supply conditions within laboratory bioprocesses. Technical setups vary from pulse-profile addition of substrate (*e.g.* described in Neubauer *et al.*[19,20]) to deliberate mixing time enhancement (*e.g.* Schilling *et al.*[21]) and compartmented reactors, which allow the investigation of several environmental inhomogeneities in parallel. Scale-down designs can be adjusted to represent mixing properties of technical scale reactors through modeling and simulation, as was shown for two-compartment reactors by Delvigne *et al.*[22], which facilitates the prediction of large-scale performance.

The significance of scale-down approaches for biotechnology is mirrored by the large number of successfully characterized metabolic properties in industrial microorganisms (*e.g.* overview in Neubauer *et al.*[17] and Takors [18]). Results clearly indicate that almost all aspects of microbial metabolism, growth and production properties are affected by bioreactor inhomogeneity. With increased understanding of metabolic effects in response to oscillations, scale-down simulation can help to identify better production strains and optimize industrial operating conditions in the future.

Surprisingly, despite its industrial importance, few publications have focused on the scale-down characteristics of *C. glutamicum*[21,23,24]. The only literature source that investigates reactor inhomogeneity is Schilling *et al.*[21], who cultivated an auxotrophic L-lysine producer in a modified stirred tank setup. This study focused on increased mixing times without further gradient characterization, thus making it rather difficult to relate the described effects, *i.e.* decrease of growth and enzyme activities, to the specific source of the disturbance. Multi-compartment reactors have not been applied so far.

We have therefore chosen to use a well characterized two-compartment reactor [25] as a promising experimental alternative. The study aims to assess the process engineering and metabolic consequences of oxygen and substrate supply oscillation for *Corynebacterium glutamicum*. Combining bioprocess engineering and distinct industrial bioreactor-like conditions with a systems biology perspective and modern bioanalytics, the effects on growth, metabolic activity, and net carbon utilization can be characterized. The controlled application of defined reactor inhomogeneity reveals metabolic and physiological effects within the separated reactor compartments, *i.e.* aerobic stirred tank reactor (STR) vs. non-aerated plug flow reactor (PFR), as well as the macroscopic effect for entire cultures of *C. glutamicum*.

## Results

### Oxygen uptake at inhomogeneous supply conditions

**Figure 1:**
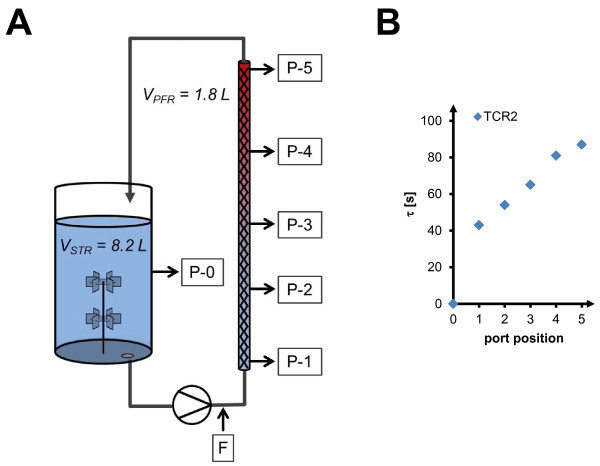
**Two-compartment scale-down reactor setup. (A)** = two-compartment scale-down reactor for analysis of oxygen supply and substrate oscillations as described in Junne *et al.*[25] and position of sampling/sensor ports (P-[0–5]), F = feed line entry, color gradient represents accumulation of fermentative by-products over anaerobic residence time (from blue to red); **(B)** = mean residence time τ at individual port positions for experimental condition TCR2.

**Figure 2:**
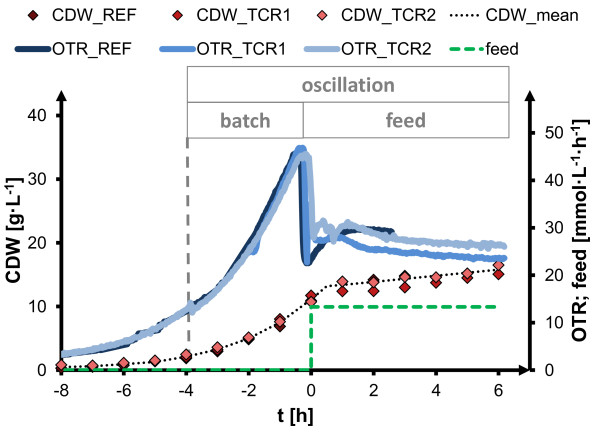
**Process overview for two-compartment scale-down cultivations of *C. glutamicum *ATCC13032.** First phase: oscillating oxygen supply at substrate excess (batch, from *t* = −4 h until *t* = 0 h); second phase: oscillating oxygen/substrate supply (feed, after *t* = 0 h); scale-down cultivations with residence time τ = 45 s (TCR1) and τ = 87 s (TCR2); reference experiment without oscillation in aerobic stirred tank (REF); cell dry weight (CDW, left axis, samples taken from stirred tank compartment), glucose feed rate (feed, right axis), oxygen transfer rate (OTR, right axis, same unit as feed); initial batch phase from t = −8 until t = 0 with 22 g/L glucose, fed-batch phase started at t = 0; dissolved oxygen (DO) levels were always > 30% for aerobic stirred tank compartment and absence of DO signals at all sensors in the plug flow compartment showed anaerobic conditions.

In order to study oscillation of oxygen supply during substrate excess, batch growth was analyzed in a two-compartment bioreactor (TCR, setup see Figure 1, process data see Figure 2). During the batch phase substrate was present in excess. Initial batch glucose concentration was 22 g/L which was completely consumed at the end of batch phase (time t = 0, Figure 2) when fed-batch phase was started. Residence times within the non-aerated plug flow compartment were set to 45 s (cultivation TCR1) and 87 s (cultivation TCR2) in different experiments, respectively. For each residence time we have performed one experimental run as a set of experiments with gradually increasing process inhomogeneity. The aim was to characterize basic principles of *C. glutamicum* in an oscillatory scale-down bioreactor environment, which can best be demonstrated with such an approach.

Anaerobic conditions appeared in the plug flow part of the reactor due to the high glucose concentration and the resulting high metabolic activity of *C. glutamicum*, which could be verified by absence of dissolved oxygen signals at all plug flow sensor ports throughout the two-compartment cultivation (Figure 1). In contrast, the aerobic stirred tank compartment was kept at high dissolved oxygen levels (DO > 30%). Reference cultivations were performed in an aerobic stirred tank without the plug flow element. After batch glucose had been used up, feed was added at the entrance of the plug flow compartment. This led to conditions of glucose excess and simultaneous oxygen limitation within the plug flow compartment, whereas the overall cultivation remained substrate-limited, and gives a lab-scale representation of the typical stress conditions in inhomogeneous fed-batch processes (Figure 2). At batch phase exponential growth and subsequent switch to substrate limited fed-batch, similar growth activity was observed at homogeneous and oscillating process conditions. From the very similar growth curve data a good reproducibility of the experiments can be deduced. Net respiration activity changed only slightly at batch conditions, as will be discussed below. This is a clear indication that cells were not affected by the substrate and oxygen oscillation, considering the balanced data of both compartments, *i.e.* the whole inhomogeneous process.

**Figure 3:**
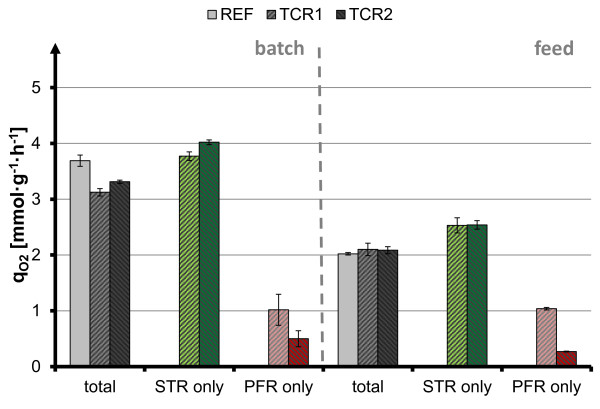
**Biomass specific oxygen uptake rate q**_**O2 **_**in batch and feed phase.** Balance-based mean uptake rate (= net uptake rate) for stirred tank compartment (STR, highlighted in green), plug flow compartment (PFR, highlighted in red) and full reactor volume (total) of *C. glutamicum* scale-down cultivation for reference experiment (REF, first bars in compartment groups), scale-down cultivation with τ = 45 s (TCR1, second bars), scale-down cultivation with τ = 87 s (TCR2, third bars), error bars indicate standard deviation over time (see Methods).

However, there is a zonal inhomogeneity of oxygen uptake between the two-compartment cultivations, which was monitored by determining the specific oxygen uptake rate (q_O2_) within the individual reactor compartments, and the whole process setup (Figure 3). Two different phenomena can be distinguished: (i) for batch conditions, *i.e.* substrate excess, the net uptake rate of the oscillating cultures is lowered by approximately 13% in comparison to the homogeneous reference cultivation, which is also the volume fraction of the PFR in relation to the total two-compartment volume. A maximum oxygen uptake rate (q_O2_) of 4 mmol g^-1^ h^-1^ is reached in the fully aerobic reference cultivation, as well as in the aerobic STR compartments of the two-compartment reactor. In contrast, oxygen supply is limited in the plug flow compartment, *i.e.* 0.5 to 1 mmol g^-1^ h^-1^ depending on anaerobic residence time. (ii) For substrate-limited feed conditions, the net oxygen uptake is identical for the two-compartment process and the homogeneous reference culture. Interestingly, the q_O2_ was higher in the STR compartment of the TCR system, compared to the reference cultivation. It seems that oxygen uptake is redistributed within the TCR, from anaerobic to aerobic zones. The q_O2_ in the PFR compartment is limiting, but is compensated for by increased uptake in the aerobic zone.

Thus, a metabolic difference in the effect of inhomogeneous oxygen supply can be demonstrated, depending on the process mode: under substrate excess batch conditions the oxygen uptake in the STR compartment reaches the maximum oxygen uptake capacity of *C. glutamicum*, and therefore is not further increased to compensate for missing oxygen uptake in the non-aerated PFR compartment. On the other hand, under substrate limited fed-batch conditions the oxygen uptake is increased to compensate for the missing oxygen uptake in the non-aerated PFR compartment.

### Metabolic impact of anaerobic residence time and glucose perturbations

**Figure 4:**
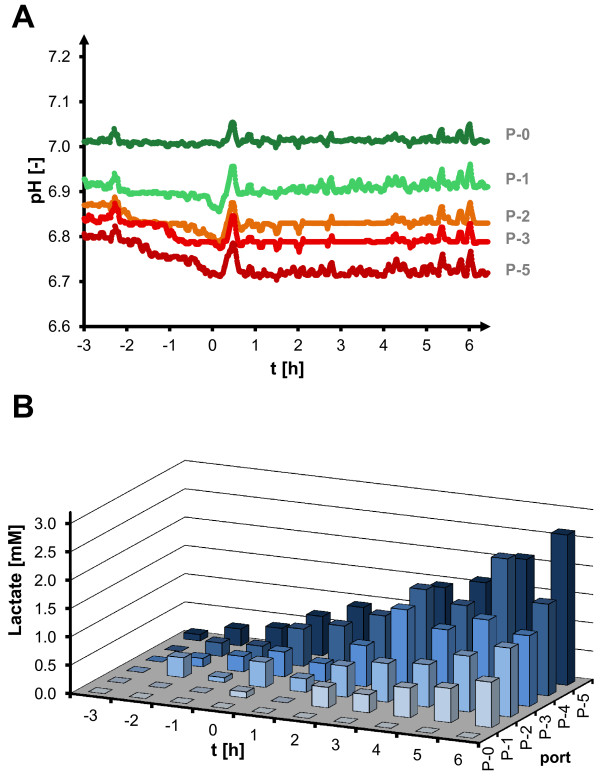
**pH (A) and lactate (B) profiles along anaerobic plug flow compartment.** Two-compartment scale-down cultivation of *C. glutamicum* for batch and feed phase; oscillation of oxygen supply (*t* < 0 h, batch) and oscillation of oxygen and substrate supply (*t* > 0 h, fed-batch) with τ = 87 s (TCR2), pH **(A)** and extracellular lactate concentration **(B)** indicated for individual port positions (P-[0–5]) as assigned in Figure 1.

Secondary metabolic effects were identified, which resulted from the perturbations in the glucose and oxygen availability in the TCR. They were monitored in the PFR compartment at the different sampling positions. Substrate uptake could be identified by decreasing glucose concentrations between plug flow ports, with a biomass-specific uptake rate of q_GLC_ = 0.90 (± 0.17) g g^-1^ h^-1^, *i.e.* 5.0 (± 1.0) mmol g^-1^ h^-1^ during the feed phase. This value is higher than the typical aerobic substrate uptake capacity of *C. glutamicum* (own data) and reported microaerobic uptake rates [26] (both *ca.* 3 mmol g^-1^ h^-1^). Interestingly, in the PFR there was also a significant pH difference between the different ports, as is shown for the longer residence time (τ = 87 s) in Figure 4A. During both the batch and the feed phase, the pH decreased along the PFR compartment. This shows that *C. glutamicum* expresses fast acidification of culture broth in response to glucose excess and oxygen depletion. However, base addition did not increase between reference and oscillating process, indicating reversibility of this acidification effect, *i.e.* formation of acids in the PFR and subsequent consumption of these acids under glucose limitation and aerobic conditions in the STR part of the TCR system.

Along with the pH drop, analysis of typical anaerobic side products of *C. glutamicum* revealed increasing extracellular lactate concentration as a major by-product over the anaerobic residence time in the PFR compartment (Figure 4B). During the feed phase, concentrations kept increasing up to a maximum of approximately 2.7 mM at the last (upper) port of the PFR compartment. However, extracellular lactate could not be detected within the aerobic stirred tank compartment. Therefore, we conclude that the lactate which accumulated under oxygen-limited conditions in the PFR part was rapidly assimilated at restored oxygen supply in the STR compartment.

**Figure 5:**
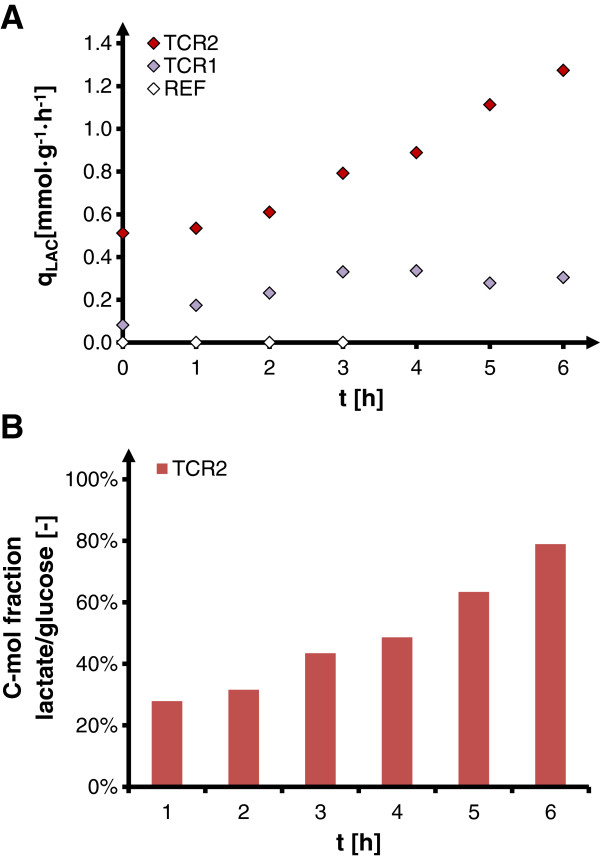
**Reversible lactate turnover at oscillating oxygen supply in two-compartment cultivation of *C. glutamicum. *(A)** = lactate turnover rates at reference cultivation of *C. glutamicum* without oscillation (REF), scale-down cultivation with short oscillations (τ = 45 s, TCR1) and scale-down cultivation with long oscillations (τ = 87 s, TCR2); lactate turnover rate q_LAC_ represents the production rate within non-aerated plug flow compartment and equals the lactate uptake rate (r_LAC,STR_) within aerobic stirred tank compartment (q_LAC,PFR_ = r_LAC,STR_) with *q*_*LAC*_ *= c*_*LAC*_ *· flow through PFR*; **(B)** = carbon fraction of feed glucose being converted into extracellular lactate [*mol*_*C_in_lactate*_*/mol*_*C_in_glucose*_] within anaerobic plug flow compartment for experimental condition TCR2.

In both experimental setups with residence times in the PFR part of 45 s (TCR1) and 87 s (TCR2), the oscillation-induced accumulation of lactate increased over the time of cultivation. The specific rate of lactate production (q_LAC_) increased with process time, reaching the highest values at long anaerobic residence times (TCR2, Figure 5A). The carbon fraction of glucose which was converted into extracellular lactate during the passage through the PFR reached considerably high values of up to 80% (Figure 5B), pointing to a double substrate process with lactate as a second major substrate at inhomogeneous process conditions. Since the lactate consumption rate in STR always equals the lactate formation rate in PFR, no net accumulation of lactate is observed in the overall TCR setup.

### Impact of oscillation on intracellular metabolite pools

**Figure 6:**
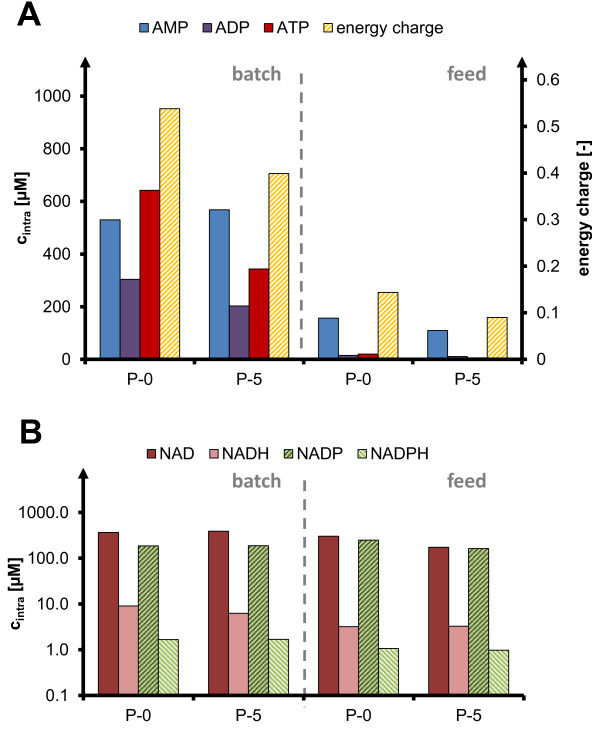
**Intracellular concentration of metabolites under aerobic conditions (P-0) and after anaerobic residence time (P-5) in two-compartment cultivation TCR2.** Quantified by metabolic quenching/LC-MS/MS [27] in batch and fed-batch phase of *C. glutamicum* cultivation with oscillation of τ = 87 s (TCR2); **(A)** = adenosine phosphates (left axis) and resulting energy charge (right axis, calculation and definition of energy charge see Methods); **(B)** = redox cofactor equivalents, batch = substrate excess and oscillation of oxygen supply, feed = substrate limitation and oscillation of both substrate and oxygen supply; AMP = adenosine monophosphate, ADP = adenosine diphosphate, ATP = adenosine triphosphate, NAD = nicotinamide adenine dinucleotide (oxidized), NADH = reduced, NADP = nicotinamide adenine dinucleotide phosphate (oxidized), NADPH = reduced.

The influence of oscillations on intracellular metabolites was analyzed after cold-methanol quenching of cell suspensions with an LC-MS/MS procedure [27], using ^13^C-labeled internal standards for absolute quantification. For this purpose, samples from the STR (P-0) and in the last (upper) port of the PFR in a two-compartment reactor cultivation with long residence time in the PFR (TCR2) were compared for the energy related adenosine phosphates (ATP, ADP, AMP) and redox related cofactors (NAD(H), NADP(H), Figure 6). Interestingly, the differences between batch and fed-batch conditions were pronounced, indicating that the extracellular glucose level is a decisive factor. For batch conditions, the pool sizes and the calculated energy charge were at least 2-3 times higher than in fed-batch conditions. In contrast, the metabolite concentrations before and after anaerobic oscillation were very similar. The energetic state of the cells seemed to be more related to glucose concentration than to the presence of oxygen. Also, no significant changes were observed for redox-related cofactors, with very similar values seen under all investigated conditions. Therefore, no pattern or trend of oscillation correlating to either glucose or oxygen presence could be identified.

## Discussion

This study illustrates the metabolic robustness of *C. glutamicum* against substrate and oxygen oscillations, which it is claimed appear within the feed zone of large-scale industrial bioreactors. It also provides insight into metabolic principles within inhomogeneous processes in general. The lack of change in growth and metabolic activity shows *C. glutamicum* possesses a high robustness against an oscillating oxygen supply, in conditions of both substrate excess and substrate limitation. Oscillations with anaerobic residence times τ of 45 or 87 s did not result in a decrease of the final biomass yield or net oxygen uptake at fed-batch conditions. Since sensitivity against oscillation has been documented in similar approaches for several other industrial organisms (*e.g. E. coli*[28], *S. cerevisiae*[29]*B. subtilis*[25], see also: Lara *et al.*[11]), this may underline the special suitability of *C. glutamicum* for large scale industrial applications, and even may explain the good practical experiences during the long history of its use in bulk chemical synthesis. Connected to this robustness, this study identified several unique metabolic properties under inhomogeneous oxygen/glucose supply.

One important difference to homogeneous cultivation is the shift of oxygen uptake from zones of limited oxygen supply to aerobic zones. This mechanism is not functional under conditions of substrate excess, where the net oxygen uptake has already reached the maximum capacity of q_O2_ = 4 mmol g^-1^ h^-1^. Under glucose limitation, however, the demand for oxygen uptake is lower, allowing a compensation of process inhomogeneity to occur. Under these conditions, a net oxygen uptake similar to that in homogeneous conditions is preserved, due to the increased uptake of oxygen within the aerobic zones of the STR. Even at longer residence times in the anaerobic part of the PFR, the relatively small volume fraction in the two-compartment system facilitates an efficient compensation by the larger aerobic bulk volume. This mechanism provides a basis for metabolic robustness against partial oxygen supply limitation, and illustrates the superiority of the fed-batch mode under inhomogeneous process conditions.

The basis for oxygen uptake compensation was identified in the rapid metabolic switch from aerobic substrate utilization to fermentative pathways. The necessity for this is evident, because substrate uptake is sustained during anaerobic oscillation. The rapid redirection of substrate carbon flow was observed directly by the increase in extracellular lactate concentrations over PFR residence time. The anaerobic metabolism in homogeneous cultures, which has already been described extensively (*e.g.* publications by Inui *et al.*[30] and Yamamoto *et al.*[31]), was shown to be rapidly activated during the anaerobic oscillations: *C. glutamicum* switches from aerobic respiration to fermentative pathways with lactate as a predominant side product among other organic acids (*e.g.* acetate, succinate). A pH decrease is a secondary effect of side product accumulation; this could also be observed in this study. The results therefore demonstrate the robustness and flexibility of the aerobic/anaerobic carbon metabolism. This is substantiated by the physiological reaction at fully anaerobic conditions, at which *C. glutamicum* undergoes growth arrest and substrate uptake decrease [26], which is not observed under oscillating conditions. It can be concluded that the physiological changes at anaerobic conditions follow a slow response, and are not triggered by short-term depletion in inhomogeneous environments.

In comparison to studies of *e.g. E. coli*[28,32], some of which made similar observations about side product turnover [33], the extent of carbon flow redirection in the different zones of the TCR is surprisingly high. Even at moderate biomass, the majority of glucose is rapidly transformed into lactate during the passage through the oxygen-limited PFR. Later, in the STR part of the TCR, lactate is metabolized; however, this kind of futile transport cycle does not affect the biomass growth. Theoretically, while lactate secretion is thermodynamically favorable, the reassimilation step should lead to metabolic energy loss (aspects on transport described in Stansen *et al.*[34]). The formation of lactate from pyruvate by lactate dehydrogenase is a reversible reaction, *i.e.* the NADH used for lactate formation is regenerated in the reaction back to pyruvate. From this point of view the intermediary formation of lactate does not consume or generate energy.

This is different for the transport of lactic acid over the cell membrane. Currently, it is not fully understood in literature how lactic acid is excreted or taken up in *C. glutamicum*. The excretion of lactic acid could be managed without use of energy, simply due to the concentration gradient under conditions of lactic acid formation. However, the following uptake (*i.e.* re-assimilation) of lactic acid against the concentration gradient would require energy to overcome the thermodynamically unfavorable concentration gradient. Assuming that one energy equivalent in form of ATP would be required for the uptake mechanism this would require one ATP equivalent for each molecule lactic acid formed during the cultivation.

Since there is no negative impact on process performance observed in this case, the required additional energy for lactate utilization seems to be negligible in the context of the overall energy metabolism. The information that there is, in fact, a high turnover of substrate into side product at oscillating oxygen supply conditions is important for the characterization of inhomogeneous processes: instead of growing on primary substrate alone, the degree of inhomogeneity defines the composition of primary substrate and secondary, organic acid carbon sources for growth in the bulk culture.

From the metabolic perspective, the rapid switch of substrate utilization during oscillating oxygen supply means that NADH reoxidation shifts from aerobic respiratory phosphorylation to detoxification through action of the enzyme lactate dehydrogenase. This should affect cellular energy levels, which are represented in the adenosine phosphates, because substrate level phosphorylation generates only minor amounts of ATP compared to the aerobic pathways. In the intracellular concentrations, however, the changes during anaerobic residence time do not seem to be of a considerable magnitude, and seem instead to be related to the level of substrate availability in the entire process. Most strikingly, the metabolism maintains its NAD(H) and NADP(H) levels throughout the oscillation phase, as the reduced side product lactate is transported out of the cells. This avoids a disturbance of the metabolic network and seems to provide a fast and flexible intermediary option for NADH reoxidation. This rapid action effectively avoids any negative effects of NADH accumulation in the cytoplasm, such as redox imbalance or decreased glycolytic substrate consumption due to reduced glyceraldehyde 3-phosphate dehydrogenase activity at an unfavorable NAD/NADH ratio. Also, the cellular energy charge (EC) is maintained at a constant level, which is in the range of previously reported studies for batch growth [5], and drops along with absolute pool sizes of adenosine phosphates at substrate limitation. This phenomenon is a typical effect of the lower substrate-to-biomass ratio, as was previously observed in other cultivations (not shown). Notably, the energy charge remains at a similar magnitude over anaerobic residence time in the feed phase, even though the cells experience substrate limitation before exposure to the oxygen supply limitation/substrate excess step change. There is, however, a generally lower pool size of adenosine phosphates observed for the substrate limited process phase, which might also influence the speed and efficiency of energy-generating reactions. In any case, the seemingly similar pool size of important metabolites during anaerobic oscillation is an indication of metabolic robustness for *C. glutamicum*.

The main advantage of the applied scale-down method, *i.e.* assessment of metabolic robustness in a scale-down bioreactor with two compartments, is that changes in microenvironment, *e.g.* pH or carbon flux redirection, can be monitored along the plug flow reactor while also checking for performance parameters of the whole process setup, *e.g.* productivity or growth. Scale-down simulators with plug flow compartments can form the link between understanding microbial responses to oscillating conditions and simulation of industrial performance. Since anaerobic residence times of 87 s are in the range of realistic mixing or homogeneity times for industrial reactors [12], this study provides an example for the assessment of microbial robustness against application-oriented process inhomogeneity. As described by Delvigne *et al.*[22], two-compartment reactors can be adjusted to mimic realistic mixing conditions of industrial reactors. With this study showing one example, a two-compartment system can also be applied for microbial robustness assessment by comparing different degrees of oscillation. In upcoming studies, this strategy can be pursued further by increasing residence times within anaerobic zones and thereby studying the metabolic robustness of *C. glutamicum* against more challenging inhomogeneities. Knowing the threshold of an organism for adaptation to oscillating conditions provides valuable insight for all process transfers, especially at large-scale industrial applications, and could give some estimation of the scalability of the particular biological system.

## Conclusions

The results of this study indicate that *Corynebacterium glutamicum* is robust against oscillating oxygen supply limitation of fed-batch environments with anaerobic residence times in the lower minute range. The reason for oxygen starvation is usually high metabolic activity, which is triggered by local substrate excess and high cell densities in large scale bioreactors. The presented scale-down approach for this phenomenon can identify metabolic properties of process inhomogeneity. The microbial response to oscillation involves a fast adaptation to the conditions in the different reactor compartments, with a high rate of lactate formation in the high glucose/low oxygen zone, and a resulting mixed-substrate uptake (joint use of glucose and lactate) in the bulk culture. The robustness of the fed-batch is mainly caused by compensation of process inhomogeneity in a rapid, reversible switch to fermentative anaerobic metabolism, which surprisingly leaves no negative impact on metabolic properties. Therefore, the native phenotype of *C. glutamicum* is well-adjusted to oscillation at typical fed-batch process conditions, which makes it particularly well-suited for large-scale application. This is unique among the industrial organisms which have previously been subjected to similar scale-down analysis. Further research should focus on the underlying physiological properties which facilitate this extraordinary robustness, in the hope of exploiting them for future bioprocess development in metabolic and process engineering.

## Methods

### Reactor setup and operation

The chosen two-compartment bioreactor setup (scale-down reactor) consists of a stirred tank bioreactor (Biostat E, Sartorius SA, Goettingen, Germany) and a connected plug flow compartment built from commercial elements, as has been previously described by Junne *et al.*[25]. It features sampling and sensor ports for pH and dissolved oxygen, both within the stirred tank and at five distinct positions within the plug flow compartment (Figure 1). The volume proportion of the two compartments is 82% for the aerobic stirred tank (8.2 L working volume) and 18% for the anaerobic plug flow compartment (1.8 L). Static mixer elements are installed along the plug flow compartment. Mean residence times *τ* at the individual ports are indicated for the experiment TCR2 with mean residence time *τ* = 87 s (Figure 1). Mean residence times were determined by extrusion experiments, as described in Levenspiel [35], and are slightly higher than hydrodynamic residence times ($\tau_{\mathrm{hyd}}=\frac{V_{\mathrm{PFR}}}{\dot{V}}$) due to backmixing effects. Plug flow characteristics were maintained at all experimental conditions, as demonstrated by the determination of Bodenstein numbers above 10 for the plug flow compartment at experimental flow conditions [29]. Due to the plug flow behavior and optimized geometry of the plug flow setup, a hypothetical impact of stagnant zones on the reactor performance can be neglected. Circulation was set to a constant flow using a peristaltic pump at flow rates of 2.64 L · min^-1^ (*τ*_*(P-5)*_ = 45 s) and 1.32 L · min^-1^ (*τ*_*(p-5)*_ = 87 s). For reference cultivation without oscillation, the plug flow compartment was omitted, resulting in a full volume of 10 L in stirred tank aerobic process with top feeding. The feed line was equipped with a backpressure valve and introduced behind the pump (reference: top feeding). Circulation through the plug flow compartment was initiated 4 h before feed start, resulting in oscillation of oxygen supply conditions at remaining batch glucose within the non-aerated plug flow compartment, before entering the feed phase with oscillation of oxygen and substrate supply. pH and dissolved oxygen sensors within the reactor setup were calibrated as described in Junne *et al.*[25]. Oxygen transfer rates (OTR) were calculated according to Junne *et al.*[36] from off-gas measurements performed with paramagnetic oxygen analyzer and spectroscopic infrared CO_2_ sensor (Binos, Fisher-Rosemount, Wessling, Germany). Biomass specific oxygen uptake rates were calculated by dividing OTR by biomass concentration, with batch phase *t* = [−2, -0.5] and feed phase t = [0.5, 2] (biomass fitted exponentially from growth curve in batch phase, linear in feed phase). Total oxygen uptake for the plug flow compartment is calculated assuming maximum solubility c_O2,max_ = 225 μmol · L^-1^ in CgXII minimal medium, as can be estimated according to media component salt effects [37,38], and assuming complete consumption from initial *c*_*init*_*= DO*_*STR*_*[%] · c*_*O2,max*_.

### Strain, media, and culture conditions

Cultivation of *C. glutamicum* ATCC13032 in the two compartment scale-down reactor was performed in CgXII minimal medium [39] containing 22 g L^-1^ of initial batch glucose · H_2_O. Constant feed was applied after the end of the batch phase (identified by increase in DO-signal) with double concentrated CgXII, 440 g L^-1^ glucose · H_2_O at a flow rate of 60 mL · h^-1^. pH was maintained at pH = 7 by addition of 25% (v/v) NH_4_OH solution. Antifoam AF204 (Sigma, Missouri, U.S.A.) was added to the medium before inoculation in 0.5‰ (v/v). Temperature was maintained at 30°C. Aeration rate was set to 0.3 vvm. In order to maintain aerobic conditions within the stirred tank, stirrer speed was regulated for DO > 30%. Reactors were inoculated with OD_600_ = 0.005 for an initial batch phase of 15 h before start of feed phase at approximately OD_600_ = 30, t = 0 h.

### Sampling

Sampling of cell free culture supernatant from ports of the stirred tank reactor and the ports along the plug flow reactor was facilitated using the self-locking Monovette® port system (Sarstedt AG, Nümbrecht, Germany) with a needle adapter remaining in every port septum throughout cultivation. Samples were taken with syringes equipped with 25 mm, 0.8 μm pore size CA-syringe filters (Carl Roth, Karlsruhe, Germany) connected to Monovette adapters. Immediate cell separation was necessary to avoid further anaerobic substrate conversion after sampling (*e.g.* lactate).

### Analytics

Cell dry weight was determined as the mean of threefold determination from cells washed in 0.9% (w/w) NaCl solution, after > 24 h of drying at 80°C. Supernatants were assayed for organic acid side products (pyruvate, succinate, malate, lactate, acetate, fumarate and citrate) with an Agilent 2100 Infinity HPLC system in 0.1 mol · L^-1^ H_2_SO_4_ at flow rate 0.5 mL min^-1^, 34 min/sample isocratic separation with column and precolumn of organic acid resin (300 × 8 mm, CS Chromatographie Service, Langerwehe, Germany). For the investigation of intracellular metabolites, the method as described in Paczia *et al.*[27] was applied, which is based on isotope dilution mass spectrometry with ^13^C-labeled internal standards from *C. glutamicum* ATCC13032 cell extracts, reaching quantitative determination after LC-ESI-MS/MS analysis. Metabolic quenching for immediate inactivation of enzymatic action was performed in a cold methanol solution, using pre-cooled syringes with 60% (V/V) methanol in 1:4 dilution (2 mL culture suspension + 6 mL pre-cooled quenching solution, resulting temperature approximately −20°C) and Monovette® port adapters, centrifugation (−20°C), and subsequent chloroform extraction from biomass (50% chloroform, 25% TE buffer, 25% methanol [V/V]). Extraction was performed in 2 mL of extraction volume with 4 h of incubation time (agitation on shaker, -20°C), centrifugation (-20°C), and aqueous phase separation. Measurement was performed as specified in Paczia *et al.*[27]. Intracellular concentrations were calculated accounting for leakage of metabolites into quenching supernatant (measured separately), and excluding metabolite leakage into culture supernatant (measured separately, leakage into culture supernatant was negligible for all presented metabolites). Cellular energy charge was calculated according to Atkinson *et al*. [23]: $\mathrm{ergy}\;\mathrm{charge}\left(\mathrm{EC}\right)=\frac{\left[\mathrm{ATP}\right]+\frac{1}{2}\left[\mathrm{ADP}\right]}{\left[\mathrm{ATP}\right]\left[\mathrm{ADP}\right]\left[\mathrm{AMP}\right]}$; (square brackets indicating intracellular concentrations).

## Competing interests

The authors have declared that no competing interest exists.

## Authors’ contributions

FK prepared the manuscript. FK and SJ designed and performed the experiments, developed and validated the experimental methods. SJ and PN designed and validated the two-compartment reactor concept. FK and SJ conducted the experiments. MO and WW initiated the project. MO is the principal investigator, and supported with design and manuscript preparation. All authors read and approved the final manuscript.
